# Aberrant expression of translationally controlled tumor protein (TCTP) can lead to radioactive susceptibility and chemosensitivity in lung cancer cells

**DOI:** 10.18632/oncotarget.21747

**Published:** 2017-10-10

**Authors:** Jiahui Du, Peng Yang, Fanhua Kong, Haiyan Liu

**Affiliations:** ^1^ Department of Thoracic Surgery, Linyi People's Hospital, Linyi, Shandong 276000, China; ^2^ Department of Thoracic Surgery, Taian City Central Hospital, Taian, Shandong 271000, China; ^3^ Department of Nursing, Linyi People's Hospital, Linyi 276000, China

**Keywords:** lung cancer, translationally controlled tumor protein, apoptosis, P53

## Abstract

Translationally controlled tumor protein (TCTP) is an evolutionally highly conserved protein which has been implicated as a biomarker for cancer cell reversion although the mechanism is not very clear. This makes it a potential target for cancer therapy. P53 tumor suppressor protein is important in regulating cell growth, it can induce either growth arrest or programmed cell death (apoptosis). TCTP and P53 has been reported that can regulate the protein level of each other.

Here we proved that TCTP is a malignancy state keeper in lung cancer and lower level of TCTP protein made cells more sensitive to stressful condition. No obvious difference has been observed from wildtype and the TCTP knockdown lung cancer cells (A549) when located in the normal circumstances. While under the stressful condition, the existence of higher protein level of TCTP can protect cells from apoptosis. TCTP and P53 formed a feedback signal pathway and through it to regulate the downstream Akt signal pathways to make the lung cancer cells keep a higher metabolism level and protect cancer cells from apoptosis induced by outside stress.

## INTRODUCTION

Lung cancer is a leading cause of mortality worldwide, which led to 1,400,000 people dead each year accounting for about 18% of all cancers. The quickness of morbidity and development of lung cancer made it important to find out biomarkers for early stage diagnosis and proper target for therapy [1].

Translationally controlled tumor protein (TCTP) is a housekeeping gene expressed in almost all the tissues including lung with a tissue specific and age specific pattern. TCTP was taken as an important target for anti-cancer drug development since its abnormal distribution pattern in many kinds of tumors [2–4]. Multiple cancer-related functions of TCTP have been reported and it is involved in the whole process of cancer such as tumorigenesis, tumor development, metastasis, and invasion etc. [3, 5]. The effect of abnormal expression of TCTP protein includes proliferation promotion [6], cell cycle protection [7], anti-apoptosis [8] and so on. According to the previous studies, TCTP is a ‘broad social’ protein. It can regulate and be regulated by lots of different factors. Thus TCTP is involved in different signal pathways and performing different functions in the processes mentioned. It is very likely that to perform one certain function, TCTP may employ a specific group of partner factors which are very different from the groups of factors employed to perform other functions. This make it difficult to figure out precisely what mechanisms and which downstream factors TCTP involved in for a certain function and in turn hamper its clinical application. That's why although drugs targeting TCTP have been already get the permission from FDA and launched [9], the follow-up report is very rare. With all the applications, precise mechanisms and signal pathways of them are still not for sure.

P53 is a famous tumor suppression protein and plays a crucial role in regulating cell growth and death. Mutations in P53 or inactivation through interaction with viral or cellular proteins are the most frequent alterations observed in cancer cells [10].

In this report, we proved that in lung cancer the negative feedback regulation between TCTP and P53 exert its function only when the tumor cells exposed to stress stimuli. Lack of TCTP protein doesn't trigger tumorigenesis initiatively. Staying in the stress-free condition, knockdown TCTP only has a very weak function on tumor cells. With the treatment of radioactivity and drugs, high TCTP protein level cells were more resistant to apoptosis and low TCTP protein level cells goes to either death or reversion. Through the direct regulatory loop, the TCTP-P53 axis has improved metabolic level of lung cancer cells exposed to stress which in turn enhancing the proliferation, metastasis and invasion in lung cells, and then resist the apoptosis induced by stress. The reciprocal regulation between the two proteins made TCTP function as a protector when cancer cells in a stressful condition. With a low level of TCTP, tumor state of the cell is more unstable and cells are more sensitive to the stress and more easily be led to apoptosis.

## RESULTS

### Translationally controlled tumor protein (TCTP) is overexpressed in human lung cancer cell both *in vivo* and *in vitro*

**Figure 1:**
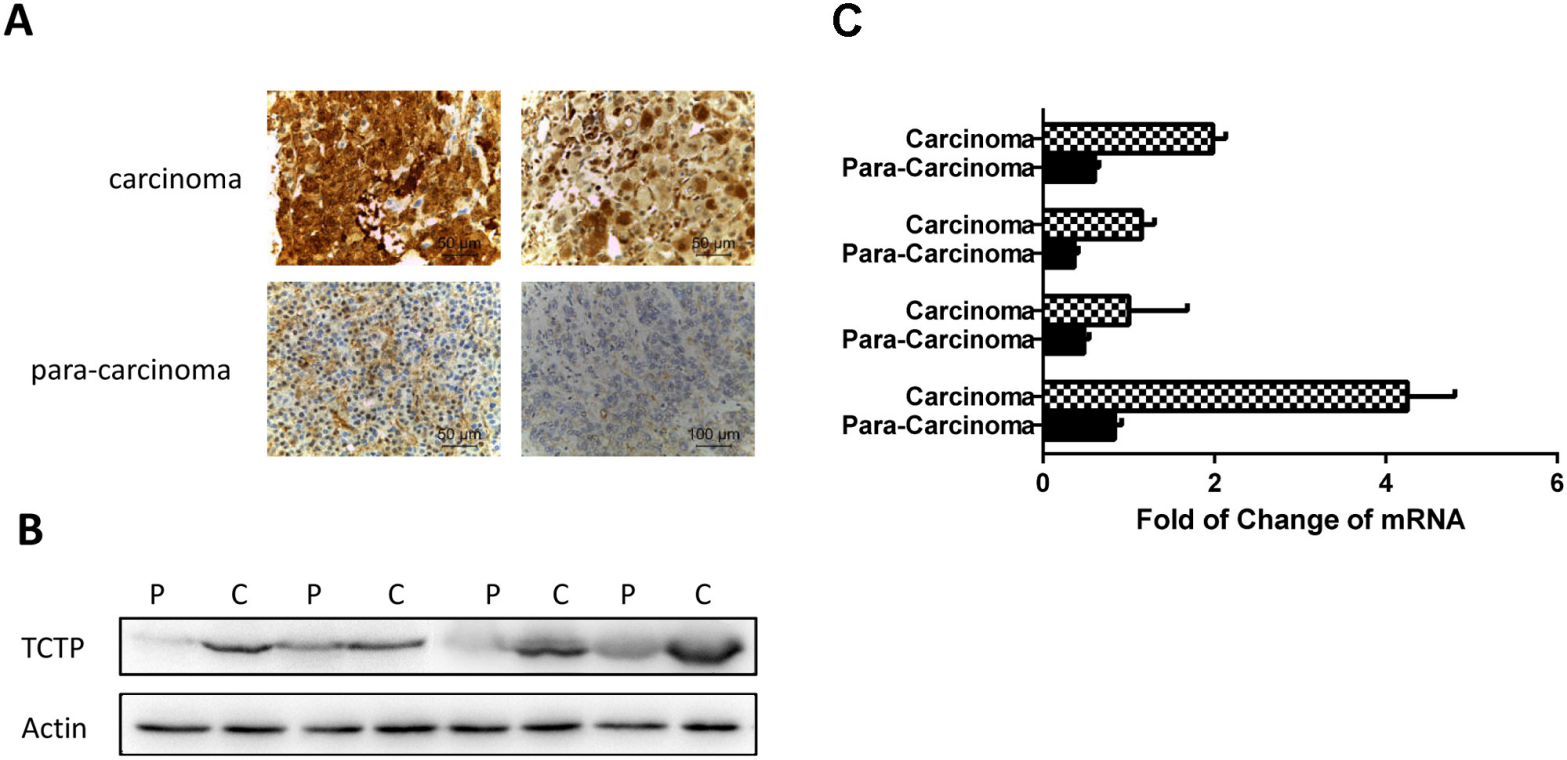
TCTP was abnormally high distributed in lung cancer compare with normal lung tissue **(A)** Two representative pairs of human lung cancers showing significant elevation of both TCTP overexpression in carcinoma tissue compare with par.a-carcinoma tissue. **(B)** Western blotting analysis of TCTP expression in multiple pairs of human lung cancer and adjacent normal tissues. c, carcinoma sample; p, para-carcinoma tissue. The data were randomly selected randomly from a large number of tissue pairs surveyed to illustrate aberrant TCTP expression in human cancers. **(C)** Q-PCR to test the TCTP mRNA in lung carcinoma and para carcinoma.

In previous studies, Translationally controlled tumor protein (TCTP) was identified as a potential lung cancer biomarkers for diagnosis by using an *in vitro* system screen [11]. To testify the consequence, immumohistochemical staining and western was taken on panel of tissues to prove the protein level in the cancer and non-cancer (Figure 1A, 1B). mRNA level in the same samples were also detected by Q-PCR (Figure 1C). Our data confirmed that TCTP was extraordinarily up-regulated in both mRNA level and protein level (Figure 1A, 1B and 1C) in multiple tumors compared to surrounding noncancerous tissues. The result strongly support the conclusion that TCTP has an abnormal high distribution in lung cancer and can be taken as a biomarker for diagnosis. Furthermore, these data also strongly implies that an important role TCTP played in lung cancer.

### TCTP knockdown alone in lung cancer cells doesn't influence reversion ratio when cell in normal condition

**Figure 2:**
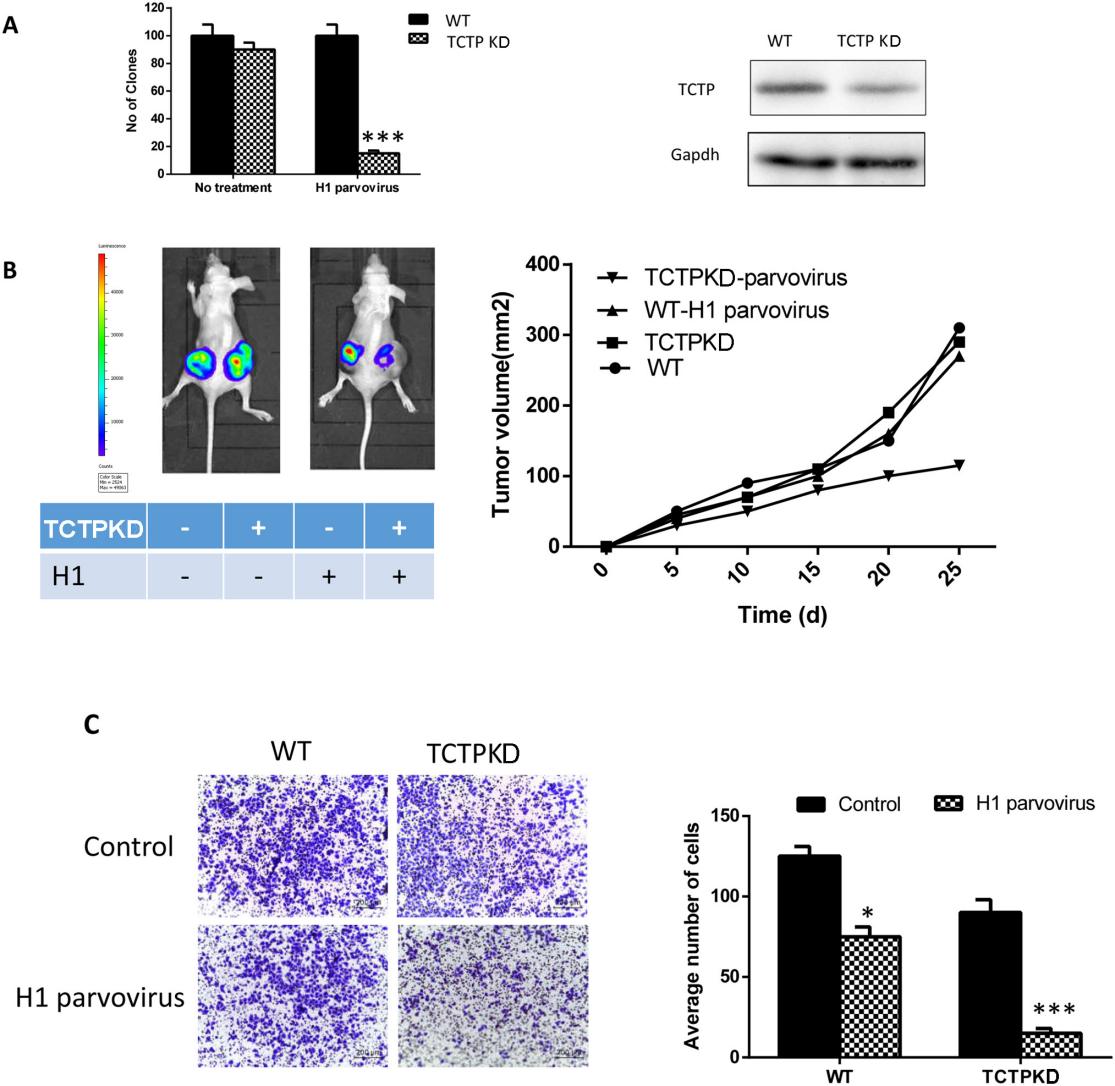
Decrease TCTP level in normal state lung cells has little effect on reversion **(A)** Knockdown TCTP in A549 with siRNA (right), the clone number in soft agar. Only under the stressful condition (transfect with H1 parvovirus), TCTP knockdown can show a remarkable effect on A549. **(B)** Tumor development in nude mouse subcutaneously injected with A549 with different treatment. Tumor formation on a pair of mice with each genotype is shown on the left, and tumor volumes (mean ± SD) from each group of six mice during the period of 6 weeks after injection are plotted on the right. **(C)** the same pattern also can be observed in transwell assay. Data are shown as mean ± SD of three independent experiments. ^*^P < 0.05, ^***^P < 0.001 compared with control.

It has been proved that when under a pressure stimuli (infected with H1 parvovirus), lung cancer cells (A549) with lower TCTP level has a higher reverting percentage [12]. To check if the same function also can be observed in lung cancer cells under normal state (non-stressful condition), we knockdown TCTP with siRNA in A549 cells and checked the clony formation of cells with different treatment through soft agar assay. For the cells treated with H1 parvovirus, knockdown TCTP lead to a dramatically decrease in number of clonies just as previous results. But for the cells without H1 parvovirus treatment (no-pressure condition), no obvious difference can be observed between the wildtype cells and the TCTP knockdown cells (Figure 2A). That is, the repression of tumor producing ability with low TCTP level only can be observed when cells under the high pressure condition. Same effect existed *in vivo*. A549 cells with different treatment were injected into the nude mice, the growth of solid tumor was repressed dramatically in the TCTP knockdown cells and treated with H1 parvovirus (Figure 2B).

Moreover, transwell assays were also proved that the difference between wild type and TCTP knockdown A549 cells depend on the condition the cells in. Without H1 parvovirus treatment, metastasis and invasion ability of A549 didn't be influenced by the TCTP level of it (Figure 2C).

Thus, We suggest that when in a no pressure condition, either the low level of TCTP protein do no harms to cancer cell physiological status or the lung tumor cells can resist negative effect caused by it, the function of TCTP is more important in a stressful condition. To prove the hypothesis, we then put the wildtype cells and TCTP knockdown A549 cells in stressful conditions which are caused by different factors, such as radioactivity or drugs and try to figure out if A549 cells with the lower protein level of TCTP are more likely to be tending to revert in all stressful conditions.

### Lung cancer cells with low level of TCTP protein are more sensitive to stressful condition caused either by chemical or physical factors

**Figure 3:**
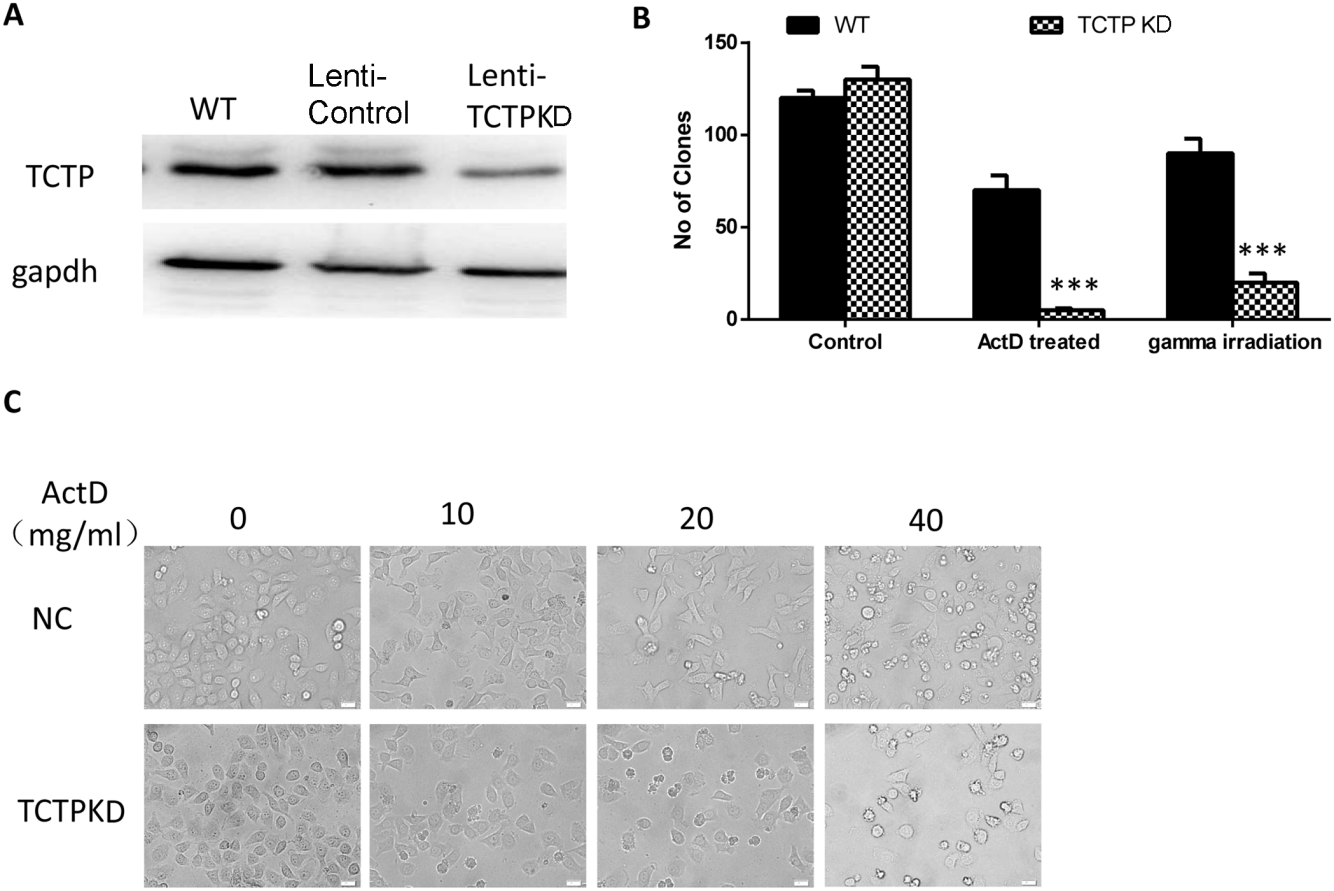
Lung cancer cells with low level of TCTP protein are more sensitive to stressful condition caused either by chemical or physical factors **(A)** Constitutive knockdown of TCTP with in A549 cells. **(B)** TCTPKO cells are more sensitive to ActD or gamma irradiation than wildtype cells. Data are shown as mean ± SD of three independent experiments. ^***^P < 0.001 compared with control. **(C)** To get the same effect on wildtype cells, compare with TCTP knockdown cells, higher concentration of ActD is needed.

We constructed TCTP constitutive knockdown A549 cells by lenti-virus. The protein levels of TCTP in A549 cells were confirmed by western blot (Figure 3A) and the proliferation of A549 doesn't influence by the infection (see Supplementary Data 1). To detect the reverting ratio alterations of lung tumor cells with different TCTP level, A549 cells (wildtype or TCKP knockdown) was treated either by ActD (10 ug/ml) (*in vitro*) or gamma irradiation (2.5 Gy) (*in vivo*). Compare with wildtype cells in non-stress condition, cells with low TCTP level showed a remarkable increase of reverting ratio in both stressful conditions just as cells infected with H1 parvovirus (Figure 3B, Figure 2C). The cells with higher TCTP level exhibited some extent of resistant to these stressful conditions (Figure 3B). Increasing the concentration of ActD, can lead to some kind of similar phenotype in wildtype cells (Figure 3D). That is, lower TCTP level caused A549 cells more sensitive to stressful conditions caused by different factors.

### A549 cells with low TCTP level was repressed in proliferation, metastasis and invasion, and easily to induce apoptosis

**Figure 4:**
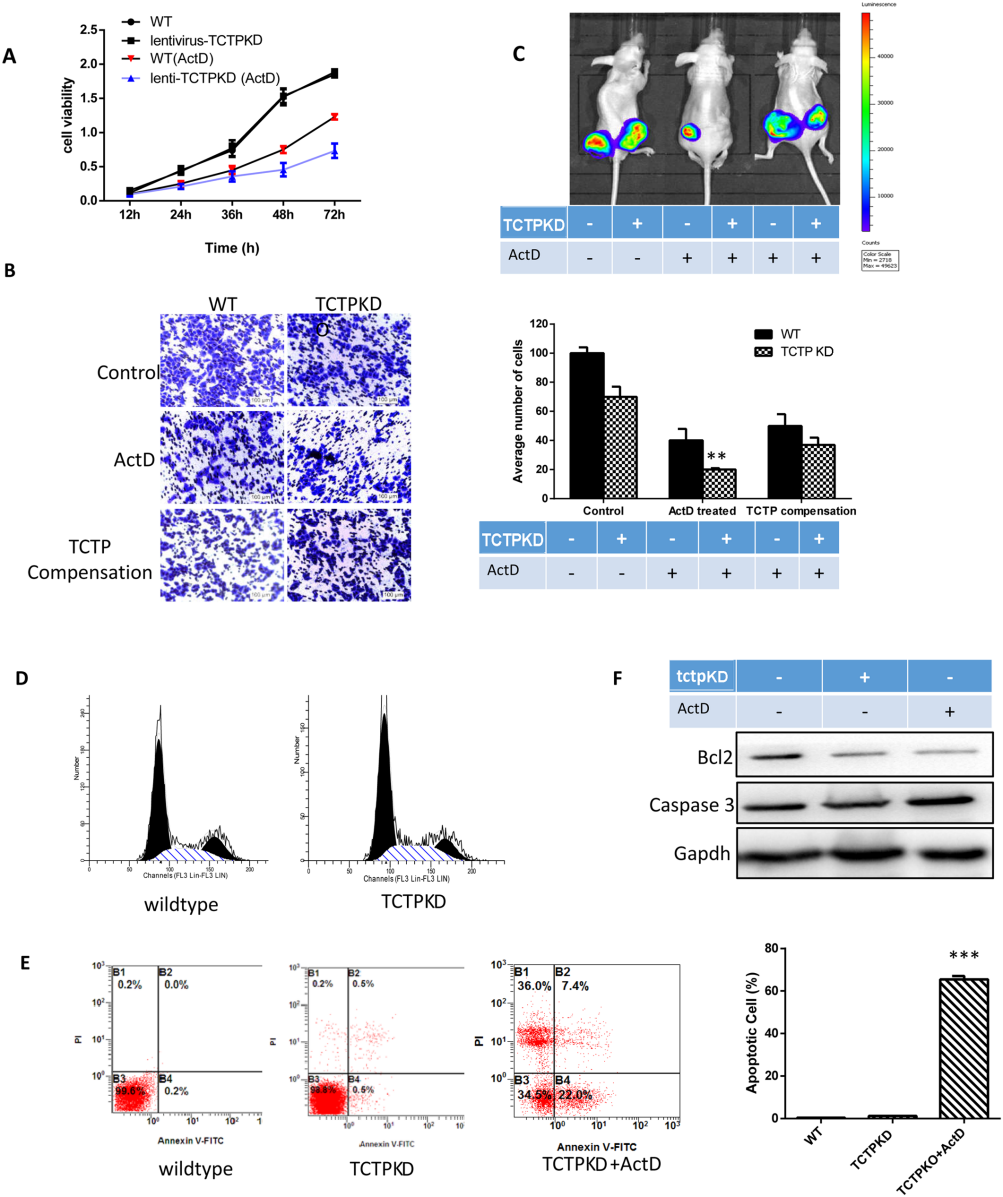
The effect of TCTP knockdown in lung cancer **(A)** Lower TCTP level caused more sever proliferation repression with ActD treatement. **(B)** ActD treatment on A549 can caused invasion repression in both wildtype and TCTP knockdown cells. Lack of TCTP leads to a more dramatic decrease and overexpress TCTP can reverse the repression. **(C)** *in vivo* tumorigenicity assay in nude mice, lower TCTP level cancer cells are sensitive to ActD treatment, the solid tumor size reduced dramatically. Overexpression TCTP to the TCTP knockdwon cells can reverse the reduction. **(D)** Knockdown TCTP in A549, cell cycle was merely influenced. **(E)** A549 cells were treated with TCTP knockdown and ActD for 24 h. Then the cells were stained with FITC-Annexin V and PI for flow cytometry analysis. The apoptotic cells were determined by the percentage of Annexin V (+)/PI (-) cells and the percentage of Annexin V (+)/PI (+)cells. Quantifcation of the flow cytometry from three independent experiments was also shown. Data are reported as mean ±SD of three independent experiments. ^**^p<0.01, ^***^p<0.001 vs. control. **(F)** TCTP knockdown doesn't induce the expression of apoptosis factor caspase3 immediately, although it indeed decrease the Bcl2 level. When under the stressful condition (ActD treatment), the caspase 3 is increased largely and the ratio of apoptosis is increased, too.

The process of tumor reversion is complicated and hundreds of genes may be involved in by activating or repressing their expression [13, 14]. To detect why TCTP can protect cells under the harmful environment and in which process of the cancer cell development the protection happened, we tested the physiological status of cells after TCTP knockdown. Compare with the wildtype, the proliferation of A549 cells was repressed dramatically with ActD treatment. Still no obvious difference was observed when the two types of cells under the non-stressful condition (Figure 4A). Furthermore, TCTP level also responsible for the tumor invasion of A549. Significant reduction (about 80%) in migration and invasion was observed in TCTP knockdown A549 with the ActD treatment (Figure 4B). Again, no remarkable difference according to TCTP level displayed in the stress less condition. The suppression was attenuated when TCTP were compensated by plasmid transfection (Figure 4B). These data clearly proved that the TCTP level in lung cancer cell A549 can influence the proliferation and migration *in vitro*. These data further proved that the protection of TCTP is essential for lung tumor development. With lower level of TCTP, the development of lung cancer are more readily controlled.

The *in vivo* tumorigenicity assay in nude mice proved the results. With the treatment of ActD before injection, both wildtype cells and TCTP knockdown A549 cells showed a remarkable reduction in tumor volume compared with the parental cells (Figure 4C).

The functional requirement of TCTP in lung cancer development is consistent with its role as a mitotic growth integrator [15], positively regulating the cell cycles. However, Flow cytometry analysis showed that cells lack of TCTP protein was only mildly arrested in G1/S phase (Figure 4D). Then, we checked the apoptosis of cells after ActD treatment. Flow cytometry analysis demonstrated that the percentage of AnnexinV-positive cells increased in cells with lower TCTP level (Figure 4E). To further verification of the easier apoptosis induction caused by the TCTP knockdown, we checked the expression level of caspase-3 and Bcl2 (Figure 4E). All the data demonstrate that other factors may involve in and through them TCTP exerts its function in the development regulation of lung cancer.

### Decrease of TCTP level lead to easier induced apoptosis and caused drug sensitive of lung cancer cells both *in vivo* and *in vitro*

Previous studies have shown that TCTP contributes to apoptosis inhibition, which protects cancer cells from immunity system attacking [16]. Furthermore, it has been reported TCTP reduced the survival rate after treatment with radiotherapy and temozolomide (RT-TMZ) for glioma patients [17]. According to our results, it is clearly at least decrease the level of TCTP increase the sensitivity of lung cancer cells to drugs and radioactivity. That is, decreasing the TCTP level of the patient may be a very helpful method to get a better effect of radiotherapy or chemotherapy. This make it very important for drugs development and personalized medicine to figure out what mechanisms involved in.

**Figure 5:**
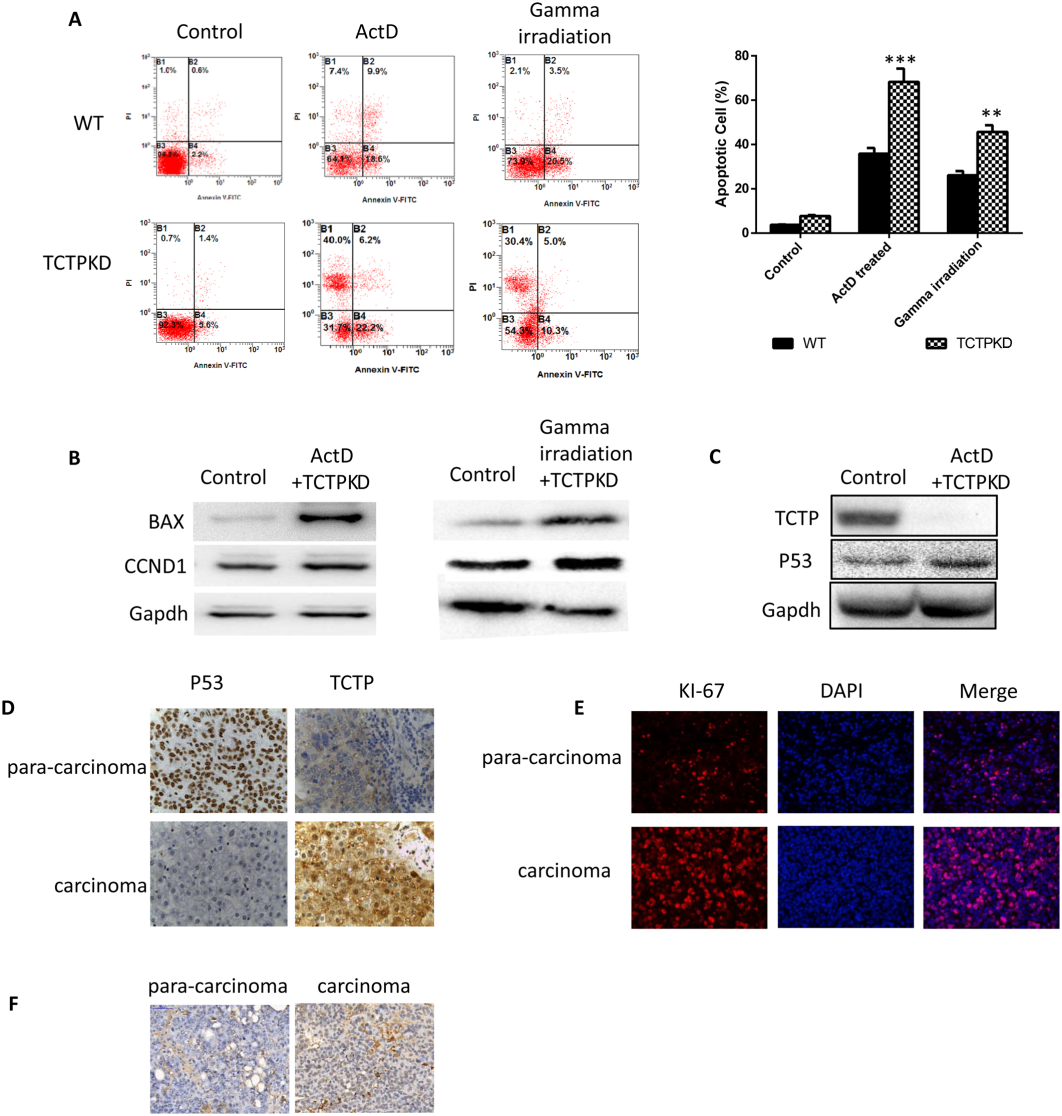
Ablation of TCTP lead to easier induced apoptosis and caused drug sensitive of liver cancer cells both *in vivo* and *in vitro* **(A)** Lower TCTP level made the apoptosis of A549 cells easier to be induced. **(B)** Apoptosis related proteins (BAX, CCND1) are upregulated dramatically in TCTPKO A549 treated with ActD or gamma irradiation. **(C)** P53 is upregulated in TCTP knockdown A549 cells with treatment of ActD. **(D)** Distribution correlation between TCTP and P53 in carcinoma and para-carcinoma tissues. **(E & F)** Low concentration of P53 and high concentration of TCTP caused high level of high proliferation (E) and low level of apoptosis (F). Data are shown as mean ± SD of three independent experiments. ^**^P < 0.01, ^***^P < 0.001 compared with control.

We therefore examined whether easier apoptosis induced by drugs and radioactivity in the low level TCTP cells are linked to the same anti-apoptosis pathway. WT and TCTP knockdown A549 cells were treated with ActD (10 ug/ml) or gamma irradiation to induce apoptosis. The same trends of apoptosis increasing can be observed by flow cytometry (Figure 5A). In both treatment, the apoptosis related proteins such as BAX, and cyclin D1 in the TCTP knockdown cells were strongly upregulated in a same pattern (Figure 5B). This indicates TCTP may employ the same pathway and mechanism to resist apoptosis induced by drugs or by radioactivity. Since cyclin D was involved in, it is reasonable to believe the apoptosis signal pathway that TCTP involved in is mitochondrion related pathway. Thus we compared the P53 expression level in the apoptosis induced wildtype cells and TCTP knockdown cells (Figure 5C). Not surprisingly, P53 level was increased dramatically in the TCTP knockdown cells. This implies that upregulation of P53 by TCTP may be involved in for the TCTP lack related sensitive. We detected TCTP and P53 expression level in pairs of lung cancer tissues and para cancer tissues by IHC (Figure 5D) and regression analysis proved the correlation between the two proteins.

### TCTP can regulate the stability of P53 in lung cancer

**Figure 6:**
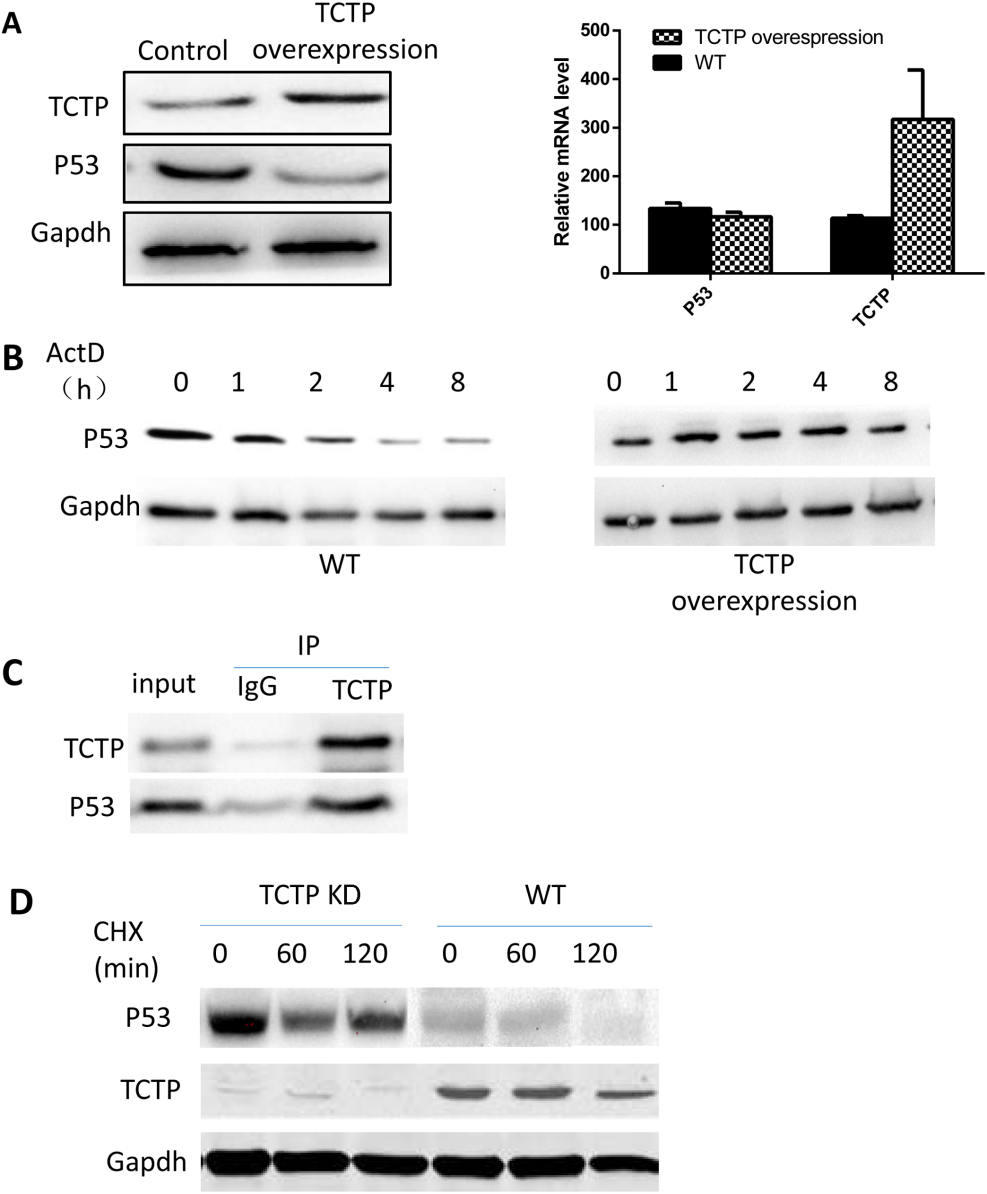
TCTP can regulate the stability of P53 in lung cancer **(A)** overexpress TCTP in A549 repressed the protein level of P53 (left) but has little effect on the P53 mRNA level (right). **(B)** Repress the mRNA synthesis of P53 and overexpress TCTP doesn't lead to P53 protein level dramatically decrease compare with wildtype A549. **(C)** co-IP proved that TCTP can bind to P53. **(D)** with treatment of CHX, knockdown TCTP can keep the protein stability of P53.

We then investigated the underlying mechanisms that TCTP regulating the P53 level in lung cancer. Overexpressing TCTP in A549 cells leads to a sharp decrease in amount of P53 protein (Figure 6A). Interestingly, the repression didn't happen in the mRNA level. mRNA level of P53 was not influenced largely by TCTP expression level. Also, inhibiting mRNA synthesis by ActD and overexpress TCTP at same time didn't caused the sharp decrease of P53 protein (Figure 6B). These data implies the regulation happened neither on mRNA transcription nor degradation. On the other side, Co-IP proved that TCTP can bind on P53 (Figure 6C). This furtherly proved that regulation is more likely happened on protein level. To prove the hypothesis, we knockdown TCTP in A549 and treated cells with Cycloheximide which can Inhibit protein synthesis. Cycloheximide treatment proved that TCTP knockdown can influence the P53 stability (Figure 6D). The protein level of P53 in TCTP knockdown A549 are much higher than wildtype. That is, interaction with TCTP can enhance the protein degradation of P53 and through this way, TCTP can regulate the P53 level in lung cancer and in turn protect cancer cells from apoptosis induced by P53.

### TCTP was regulated by P53

**Figure 7:**
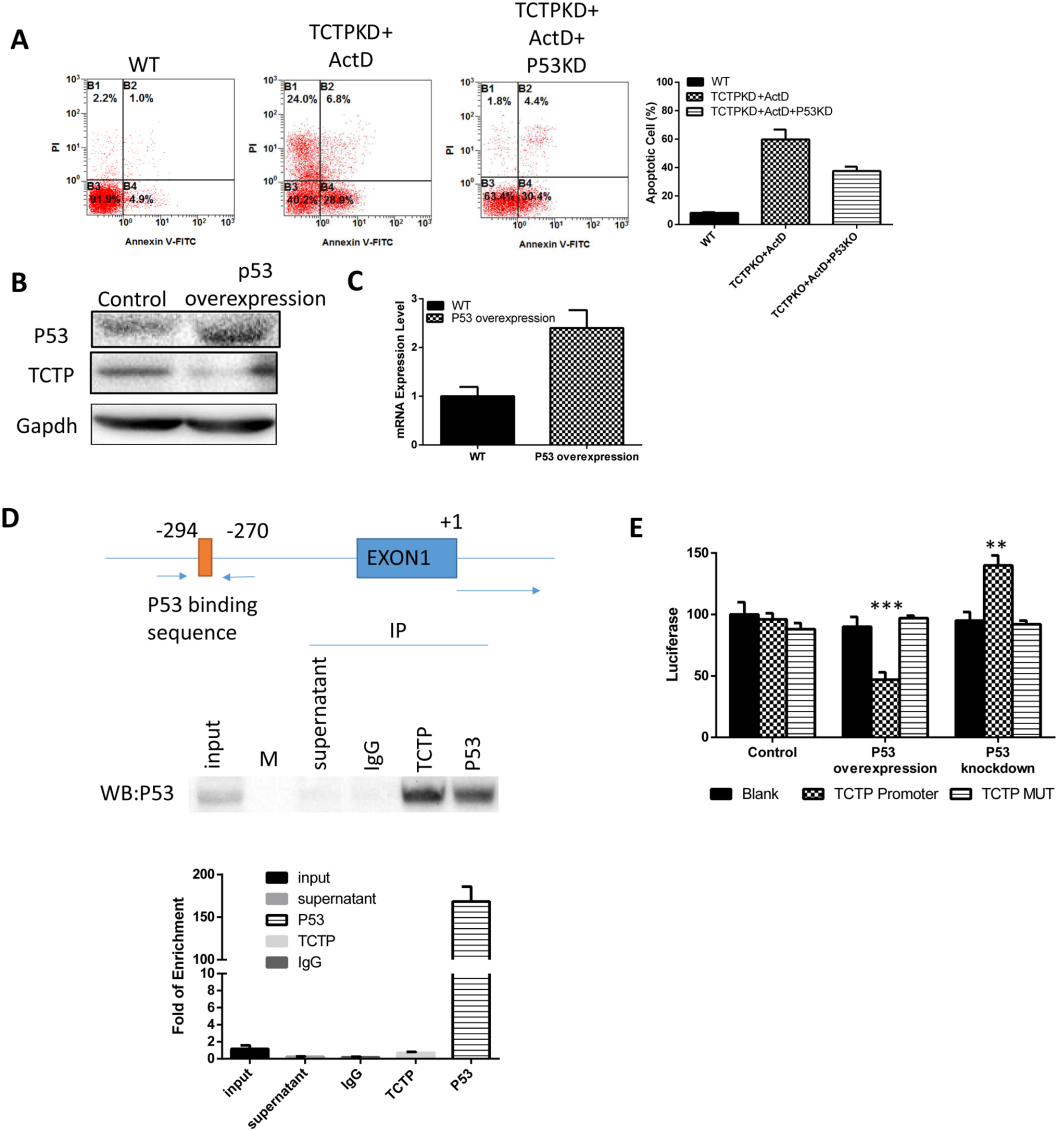
TCTP was regulated by P53 **(A)** Knockdown P53 together with P53 can partly reverse the effect caused by TCTP knockdown. **(B)** Overexpress P53 can downregulate TCTP protein level. **(C)** Overexpress P53 can downregulate TCTP mRNA level. **(D)** At the top is a schematic representation of the P53 binding sequence in the TCTP promoter; at the middle is the western results of RIP assay to prove the antibody can pulldown P53 and the mRNA it bound. Bottom is the QPCR results to testify the binding sequence. **(E)** Luciferase assay to test the P53 binding sequence in TCTP promoter and its mutant.P53 was either knockdown or overexpressed and check its effect on luciferase activity. Data represent the mean ± s.d. of 3 independent experiments. ^***^P ≤ 0.0001 ^**^P≤ 0.001.

When knockdown P53 in A549, the effect of TCTP knockdown, including easier induced apoptosis and sensitivity to drugs is partly reduced (Figure 7A). This is consistent with previous research results that P53 can enhance the apoptosis of tumor cells. Interestingly, the overexpression of P53 is accompanied with dramatically decrease of TCTP level (Figure 7B). The results strongly implies that P53 can also regulate the TCTP level in lung cancer. It has been reported that TCTP is a transcriptional regulatory factor and can regulate the transcription of set of apoptosis genes, such as Fas [18], IGF-BP3 [19], BAX [20] and so on. To prove if TCTP is one of the regulated genes of P53 in lung cancer, we detected the mRNA level of TCTP and found it is just decreased just as the protein level. That is, P53 can regulate the TCTP mRNA transcription (Figure 7C). Combining RNA Pulldown with RT-PCR, we figured out the binding sequence of P53 on TCTP promoter (Figure 7D). The luciferase Reporter with the promoter sequence was constructed and transfected to A549 to confirm the function of the sequence. The results proved with P53 can repress the TCTP mRNA transcription by binding on its promoter (Figure 7E).

### TCTP-P53 involved in Akt and Erk signal pathway

**Figure 8:**
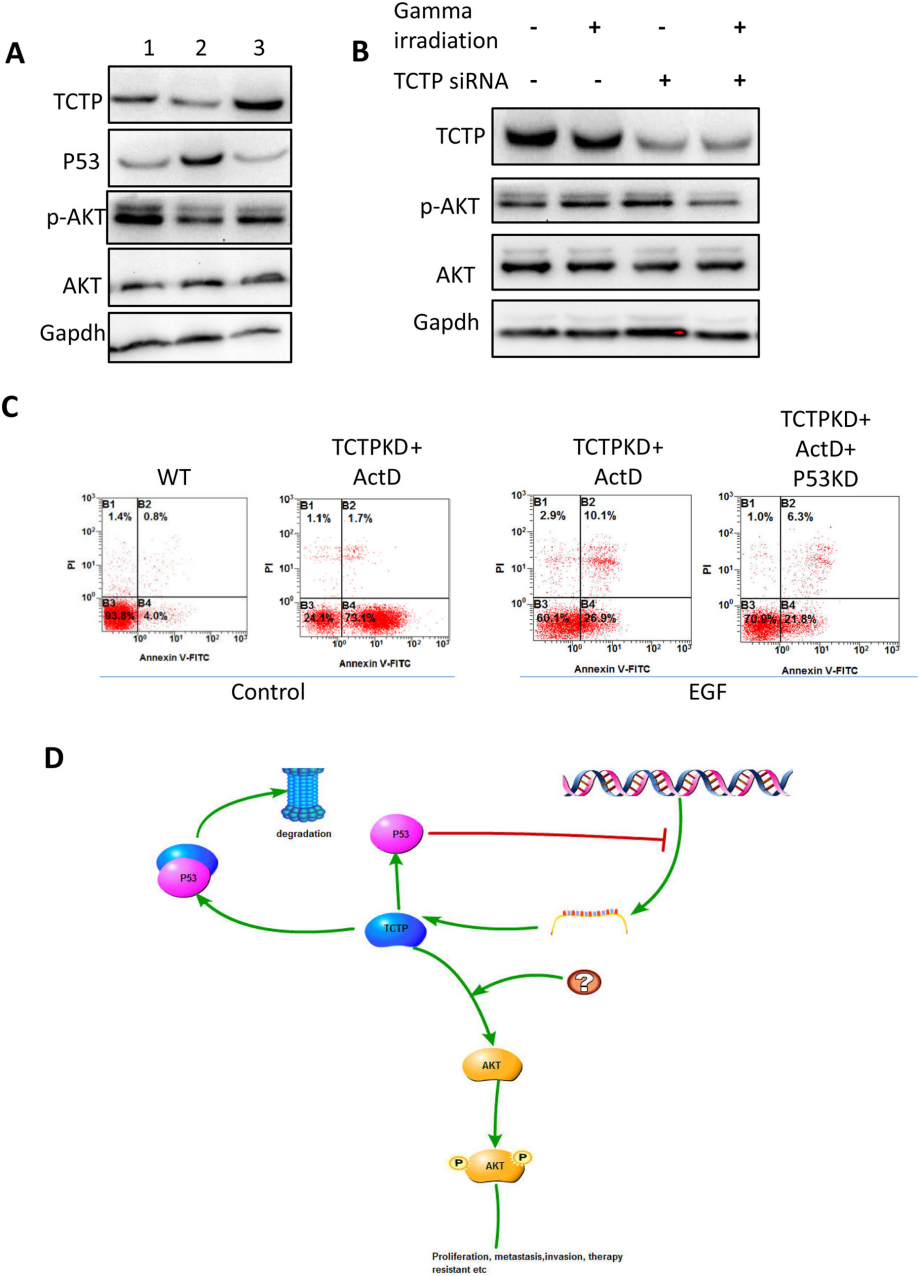
Signal pathways may involve in and the hypothesis **(A)** Compare with wildtype A549 cells (1), overexpress P53 (2) leads to decrease level of p-AKT. The repression was relieved with TCTP overexpression. **(B)** The phosphorylation of AKT was decreased in the TCTP knockdown cells with gamma irradiation. **(C)** Activate AKT with EGF, the sensitivity to ActD in TCTP knockdown cells was repressed and knockdown TCTP didn't reverse the effect. **(D)** Hypothesis of P53-TCTP feedback signal pathway in lung cancer.

We further tried to identify how P53-TCTP axis would regulate the tumor metastasis and keep the tumor cell state. Because both P53 and TCTP were critically contributing to the tumor cell proliferation and influence the apoptosis. It is reasonable to suspect that Akt pathway been involved in. We then checked the Phosphorylation pattern of Akt in A549. Overexpression of P53 can repress the Phosphorylation and overexpression of TCTP can partly remove the repression (Figure 8A). Interesting thing is, overexpression TCTP doesn't give such remarkable effect on either Akt expression or AKT phosphorylation. The phosphorylation repression happened when the TCTP knockdown cells treated with gamma irradiation (Figure 8B). Activating Akt phosphorylation by treating cells with EGF, the sensitive induced by TCTP knockdown was relieved obviously. And knockdown P53 cannot reverse the phenomena (Figure 8C).

Here, we draw a conclusion, that TCTP-P53 axis was activated by signals from stressful condition and partly exert downstream regulation including tumor cell proliferation, apoptosis and so on through PI3K/Akt signal pathway ton (Figure 8D).

## DISCUSSION

Remarkably overexpressed of TCTP protein has been observed in lung cancer and it has been considered as a critical biomarker for early stage diagnosis [11]. One of the interesting feature of TCTP in cancer is, it has been identified as a biomarker of malignancy reversion in some kind of cancers including lung [12]. In some of the tumors, repressing the expression of TCTP protein alone can make the cancer cells lost their malignancy features and revert to normal cells.

In this study, we found that in the lung cancer, although repress the TCTP protein level in the revertant potential cancer cells can increase the reverting ratio, knockdown TCTP alone in the lung cancer cells didn't show such big difference. But when being in the stressful condition such as virus infection, radioactivity or drugs, TCTP protein level can decide the destiny of lung cancer cells. Lower TCTP level made cells more sensitive to the condition stress and easier to be induced to apoptosis. That is, the TCTP levels in the tumor cell may decide the effect of radiotherapy and chemotherapy to lung cancer. Thus mechanisms of TCTP employed is important both for theory research and clinical lung cancer therapy.

A negative feedback regulatory loop between P53 and TCTP previously have been reported [21]. TCTP controls the protein stability of P53 [22], while P53 repress the TCTP mRNA transcription [23]. Our data proved that in the lung cancer cells, the negative feedback loop also worked the same way: overexpressed P53 lead a lower TCTP level by repressing TCTP mRNA transcription, which in turn make the inactivation of Akt pathway and caused a series of events during the cancer development including: proliferation repression cell cycle arrest and apoptosis ratio increasing. It's not surprisingly that higher level of TCTP can resist these P53 induced effect. But knockdown TCTP only didn't lead to P53 overexpression in lung cancer, although theoretically, lower TCTP level can make P53 protein escaping from degradation induction. The downstream effect of P53 was not observed in TCTP knockdown in A549 cells either. The effect of TCTP knockdown can only be observed in A549 cells when it is in a stressful condition. Lower TCTP level cause the lung cancer cells more sensitive to the harmful factors and easier to be induced to apoptosis. Compared with TCTP knockdown cells, wildtype A549 cells, which have a higher TCTP level, show a remarkable resistant to all harmful conditions and more likely to survive in the condition. Overexpression of P53 can partially reduce the resistant, while knockdown TCTP gives a more severe sensitivity to the lung cancer cells, which means other factors or signal pathways may also be involved in the process.

By proving the reciprocal regulation between P53 and TCTP play an important role to influence the cell sensibility to stressful circumstance in lung cancer and figure out at least part of the section in the mechanism which decide the effect of cancer therapy, our study points out a pivotal role of TCTP in lung cancer with potentially important clinical and therapeutic implications.

## MATERIALS AND METHODS

### Cell line

A549 cells were maintained in Dulbecco's modified Eagle medium supplemented with 10% fetal calf serum. The both siRNA and plasmid transfection were following the standard protocol with Lipofectamine 3000. siRNAs were synthesized by Gene Pharma Co. Sequences of siRNAs used can be found in Supplementary Data 2.

### Revertant cells

The revertant A549 cells were get as described [12]. H1 parvovirus were used to infect A549 with a concentration of 100 plaque-forming units per cell. Surviving colonies were isolated by using collagenase/dispase (Roche Diagnostics). Growth of revertant cells and wildtype cells was tested in soft agar (agar-noble, Difco). For the *in vivo* test, nude mice were injected with 10^7^ wildtype cells or revertant cells per mouse, and statistical analysis on the growth was performed. To test the infection ratio, H1 parvovirus DNA was amplified by using the following primers: 5’-CTAGCAACTCTGCTGAAGGAACTC-3’ and 5’-TAGTGATGCTGTTGCTGTATCTGATG-3’.

### Firefly luciferase assay

To validate the transcriptional regulation of P53 to TCTP, A549 cells were seeded at a density of 3 × 10^5^/ml in a 24-well plate and treated with ActD (10 ug/ml) after attachment (4 hours after seeding) if needed. 24 h later, plasmids constructed by psiCHECK™-1 Vector (Promega) inserted with UTR sequence of TCTP (nucleotides –880/+300) or control sequence was transfected. 24 hours later the cells were then lysed and the luciferase activity was measured using luminometer (T20/20, Promega).

### Western blot

For the cells, harvested and were lysed in RIPA lysis buffer with proteinease inhibitor cocktails (Beyotime Biotechnology, China) at 4 degree for 10 mins. For tissues, the small slides of tissue was put in the RIPA lysis buffer with proteinase inhibitor cocktails (Beyotime Biotechnology, China) and lysis with ultrasonication. The supernatants were collected and the protein concentrations were determined using a BCA protein assay kit (Beyotime Biotechnology, China). Total protein extracts were separated by 12% SDS-PAGE and transferred to polyvinylidene difluoride membranes. The membranes were incubated with primary antibodies, including anti-TCTP (Cell Signaling Technology CST, Danvers, MA, USA), anti-AKT (Cell Signaling Technology CST, Danvers, MA, USA), anti-p-AKT (CST), anti-P53 (CST), and anti-actin (Abcam, Cambridge, UK), overnight at 4°C. After the membranes were incubated with horseradish peroxidase (HRP)-conjugated secondary antibodies (Abcam) for 1 h at room temperature, the protein bands were detected with a ChemiDocTM XRS+ and Image Lab TM software (Bio-Rad, Hercules, CA, USA).

### Co-immunoprecipitation (Co-IP)

A549 cells were lysed with an ice-cold lysis buffer (10 mM HEPES [pH 7.2], 142.5 mM KCl, 5 mM MgCl2, 1 mM EGTA, 0.2%NP-40, 1 mM phenylmethylsulfonyl fluoride, 5 ug of leupeptin/ml, and 10 ug of aprotinin/ml). Precleared cell lysates were then incubated with protein A-agarose beads (Amersham) linked with antibodies of anti-TCTP, anti-P53 (CST), or the control antibody. The immunoprecipitated protein complexes were resolved by SDS-polyacrylamide gel electrophoresis (PAGE) and transferred to the polyvinylidene difluoride membrane (Millipore), and the coimmunoprecipitated proteins were analyzed by immunoblotting with anti-TCTP (rabbit source), anti-TCTP (rabbit source), or other antibodies as indicated in the figures. After probing with an appropriate horseradish peroxidase-conjugated secondary antibody, specific signals on the membrane were visualized by using an enhanced chemiluminescence system (Amersham) according to the manufacturer's instructions.

### RNA binding protein immunoprecipitation (RIP)

5 ug Anti-P53 antibody (CST) was incubated with 50 ul beads on ice for 30 minutes. 1.2^*^108 A549 cells was lysis by RIP lysis buffer and incubate with antibody treated beads on ice overnight. Treat the immunoprecipitation product with the diluted RNase as described in protocol. RNA was extracted from the immunoprecipitation product and reversion transcription was taken to get the cDNA. Amplify the promoter sequences by PCR and the product was sequenced to prove the binding region.

### Quantitative PCR assay

The total RNA from the transfected cells was extracted using a one-step extraction method with TRIzol (Invitrogen). To assess the RNA quantity and quality, absorbance at 260 nm and 280 nm was measured using a microplate reader (Epoch, BioTek). For each sample, 500 ng of RNA was used as the template in a 10-μl cDNA transcription system. A standard reverse-transcription (RT) protocol supplied by Promega (utilizing MMLV) was used with oligo(T)18. This procedure was followed by PCR amplification and product separation on 2% agarose gels or Q-PCR amplification using an IQ5 (Bio-Rad). In all the PCR or Q-PCR systems, 1 μl of cDNA was used as the template. Gel images were captured using a GeneGenius Gel Imaging System (SynGene) and quantified with the associated GeneTools analysis software. The primer sequences can be found in Supplementary Data 2.

### Immunohistochemistry

Tissue samples were fixed in formalin, embedded in paraffin and sectioned to 4-μm thickness. The sections were deparaffinized, hydrated, and boiled in 10 mM citrate buffer (pH 6.0) for antigen retrieval. Endogenous peroxidases were inactivated with 3% H_2_O_2_. After the sections were blocked in goat serum, the sections were incubated with primary antibody (anti-TCTP or anti-P53, CST)

overnight at 4°C, incubated with biotinylated secondary antibody at room temperature for 1 h, and visualized with diaminobenzidine (DAB kit, ZSGB-BIO, China). Hematoxylin was used to counterstain the nuclei.

### Flow cytometric assay to detect the cell cycle and apoptosis

A total of 1×10^5^ A549 cells were seeded into 6-well plates and treated with ActD (10 ug/ml) after attachment (4 hours after seeding) if needed. Then the cells was transfected with siRNA or plasmids with lipofectaime3000 followed protocol. Culture medium was changed 4 hours after transfection. 24 hours later, the cells were then collected and fixed with 75% cold alcohol (for cell cycle detection) or just collected with PBS (for apoptosis detection). Then, the cells were centrifuged at 1,000 x g for 5 min. The cell cycle stage of the tumor cells was measured by flow cytometry (FACSAria; BD Biosciences) at 488 nm. The apoptosis samples were dyed with fluorescein isothiocyanate (FITC)-Annexin V was measured at 520 nm.

### Transwell

A total of 1×10^4^ A549 cells was suspended in 200ul of 0.1% bovine serum albumin medium. The suspensions were seeded into the transwell chambers (8 μm pore size; Millipore) which was treated with or without Matrigel (BD Biosciences, San Jose, CA, USA), 600 μl complete medium was added to the bottom chamber and cells were cultured with 5% CO_2_ at 37°C for 24 h. Then, the cells were washed three times with cold phosphate-buffered saline, fixed, stained. The top surface was wiped off by cotton swab. Cells adhering to the bottom surface of the membrane were counted in fve randomly selected areas under a 200× microscope feld. Each experiment was repeated three times.

### CCK8

A549 Cells were cultured into 96-well cell culture plates with a concentration 5^*^10^3^/well and permitted to adhere for 12h at 37°C. Cells were washed three times with ice cold PBS and treated with ActD (10 ug/ml) for 24 h at 37°C. Cell viability was assessed using the Cell Counting Kit 8 (CCK-8, Yiyuan, Guangzhou, China), according to the supplier recommendations. Cell viability was expressed as the percentage of viable cells relative to untreated cells using the absorbance at 450 nm. All experiments were performed in triplicate on three separate occasions.

Each experiment mentioned in the paper repeated at least three times to ensure the certainty.

## SUPPLEMENTARY MATERIALS FIGURES



## Abbreviations

lTCTP: translationally controlled tumor protein
ActD: actinomycin D
KD: knockdown
