# Supplementary material for: Aberrant expression of translationally controlled tumor protein (TCTP) can lead to radioactive susceptibility and chemosensitivity in lung cancer cells
Source: Oncotarget. 2017 Oct 10;8(60):101922–35. doi: 10.18632/oncotarget.21747 (PMC5731924; doi:10.18632/oncotarget.21747)
Supplement: Supplementary file 1 [file oncotarget-08-101922-s001.pdf]

Aberrant expression of translationally controlled tumor protein (TCTP) can lead to radioactive susceptibility and chemosensitivity in lung cancer cells

SUPPLEMENTARY MATERIALS

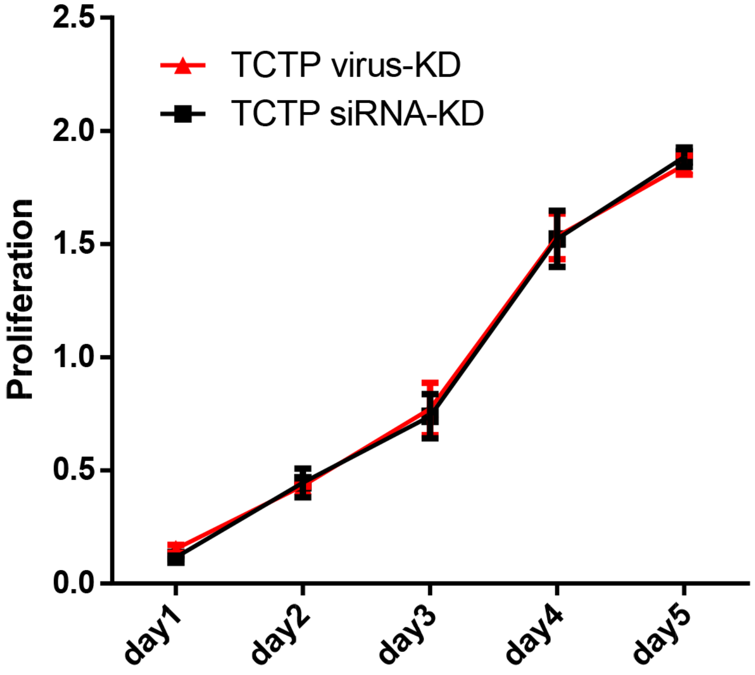

Supplementary Data 1: TCTP knockdown with siRNA or with virus made no significant difference in cell proliferation.

A siRNA Sequenc List

UPF1 siRNA: 5'-CCUUCCCAUCCAACAUCUUTT
TCTP siRNA: 5'-UCUCCGACAUCUACAAGAUTT
P53 siRNA: 5'-GAAGAAAAUUUCCGCUUUUTT

B Primer sequence List

TCTP-F AGACCAGAAAGAGTAAAA
TCTP-R TCCACTCCAAATAAATCACAG
GAPDH-F GCACCGTCAAGGCTGAGAAC
GAPDH-R TGGTGAAGACGCCAGTGGA

Supplementary Data 2: all the sequences used
